# Supplementary material for: Asymmetric Conservation within Pairs of Co-Occurred Motifs Mediates Weak Direct Binding of Transcription Factors in ChIP-Seq Data
Source: Int J Mol Sci. 2020 Aug 21;21(17):6023. doi: 10.3390/ijms21176023 (PMC7504069; doi:10.3390/ijms21176023)
Supplement: Supplementary file 1 [file ijms-21-06023-s001.zip › ijms-894674 suppl final/ijms-894674 Figures S1-2.pdf]

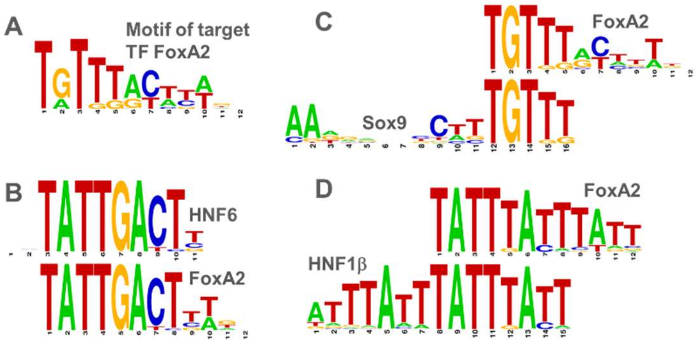

Figure S1. The most common structural variants of asymmetric CEs with more conserved motifs of partner TFs HNF6 (B), Sox9 (C) and HNF1β (D). Panel (A) shows the motif of the anchor TF FoxA2, reveled in de novo motif search [8] for the ChIP-seq dataset [15].

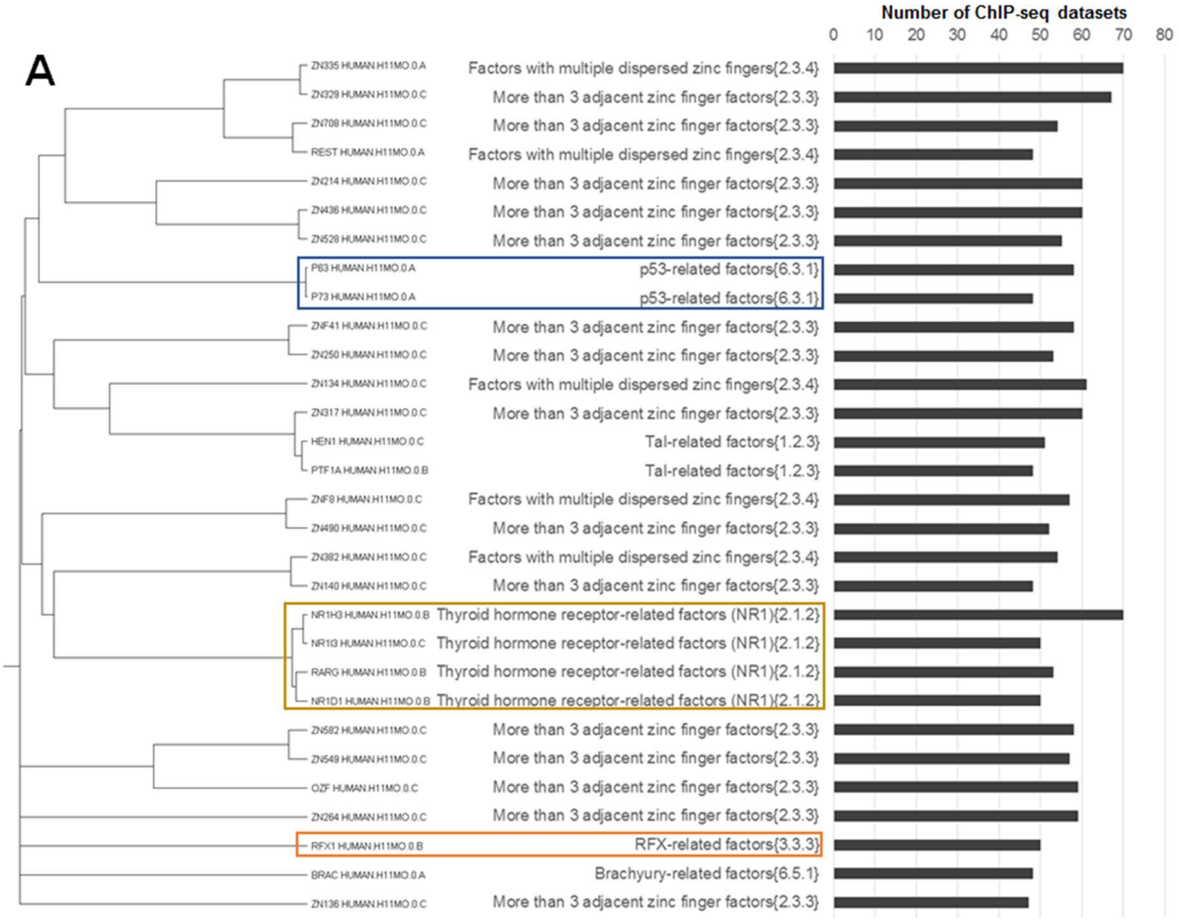

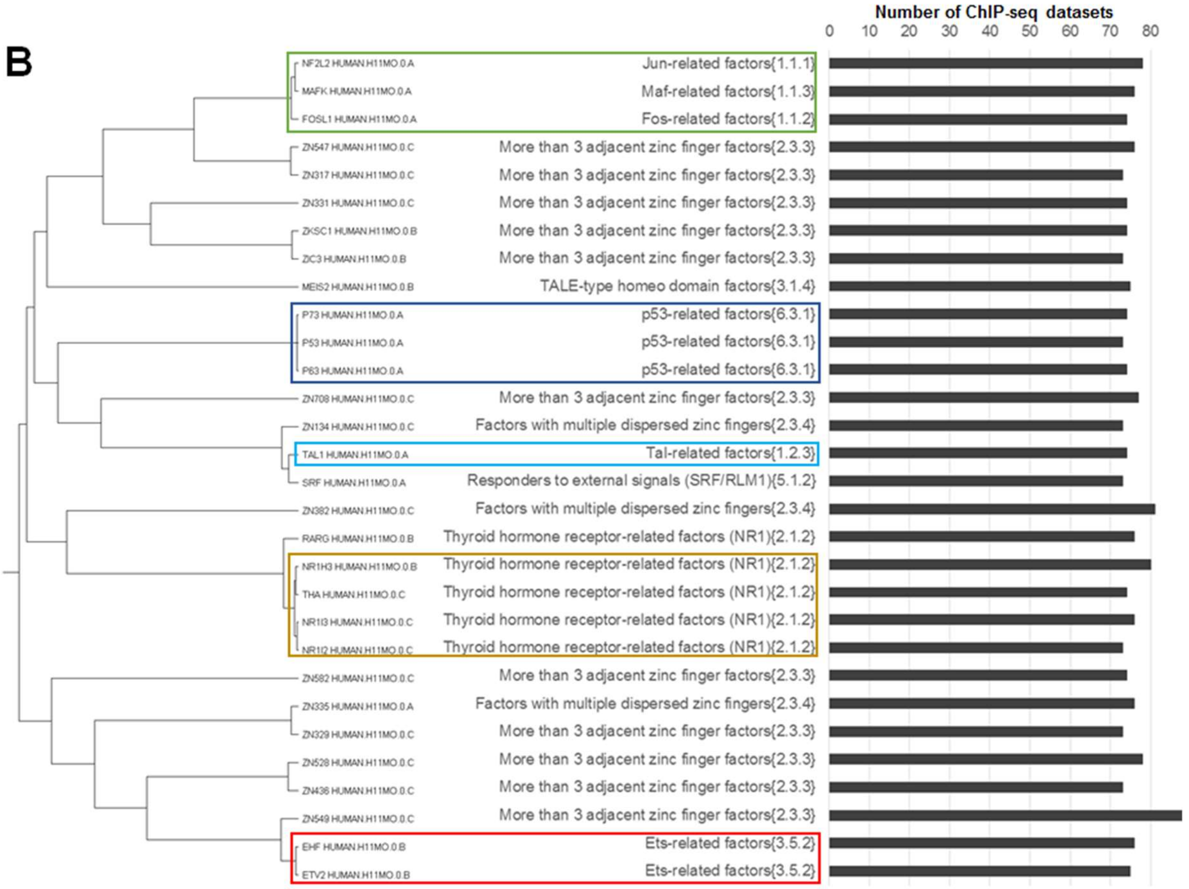

Figure S2. Clustering of 30 top-ranked partner motifs from the Hocomoco human core collection [14] according to their abundance in CEs predicted with an overlap of anchor motifs. We left in analysis CEs containing the significant homology between an anchor and partner motifs. Panels A and B show results for CEs with more conserved anchor and partner motifs, respectively. For each panel the left/middle/right columns show the tree constructed according to motifs homology, names of TF families [13] and the distribution of the number of ChIP-seq datasets that contained respective significant CEs. Brown, green, red, orange, blue and aqua boxes mark NR1H3-like motifs from Thyroid hormone receptor-related factors (NR1){2.1.2} family, Jun-like (Jun-related factors{1.1.1}, Fos-related factors{1.1.2} and Maf-related factors{1.1.3}), Ets-like (Ets-related factors{3.5.2}), RFX-like (RFX-related factors{3.3.3}, p53-like (p53-related factors{6.3.1}) and GATA-like (Tal-related factors{1.2.3}) motifs, respectively. Totally, we included in analysis 119 ChIP-seq datasets for human TFs (see Materials and Methods).
